# Pervasive Hydrothermal Events Associated with Large Igneous Provinces Documented by the Columbia River Basaltic Province

**DOI:** 10.1038/s41598-020-67226-9

**Published:** 2020-06-23

**Authors:** I. N Bindeman, N. D. Greber, O. E. Melnik, A. S. Artyomova, I. S. Utkin, L. Karlstrom, D. P. Colón

**Affiliations:** 1 Earth Sciences, University of Oregon, Eugene, OR USA; 2 Department of Earth Sciences, University of Geneva, Geneva, Switzerland; 3 Fersman Mineralogical Museum, Moscow, Russia; 4 Institute of Geological Sciences, University of Bern, Bern, Switzerland; 5 Institute of Mechanics, Moscow State University, Moscow, Russia

**Keywords:** Geochemistry, Hydrogeology, Petrology, Volcanology, Climate change

## Abstract

The degree and extent of crustal hydrothermal alteration related to the eruption of large igneous provinces is poorly known and not easily recognizable in the field. We here report a new δ^18^O dataset for dikes and lavas from the Columbia River Basalt Group (16–15 Ma) in the western USA, and document that dikes on average are 1–2‰ more depleted in δ^18^O than basalt flows. We show that this observation is best explained with the involvement of heated meteoric  waters during their cooling in the crust. The largest 6–8‰ depletion is found around and inside a 10 m-thick feeder dike that intruded the 125 Ma Wallowa tonalitic batholith. This dike likely operated as a magma conduit for 4–7 years, based on the extent of heating and melting its host rocks. We show that this dike also created a hydrothermal system around its contacts extending up to 100 m into the surrounding bedrock. A model that considers (a) hydrothermal circulation around the dike, (b) magma flow and (c) oxygen isotope exchange rates, suggests that the hydrothermal system operated for ~150 years after the cessation of magma flow. In agreement with a previously published (U-Th)/He thermochronology profile, our model shows that rocks 100 m away from such a dike can be hydrothermally altered. Collectively, our sample set is the first documentation of the widespread hydrothermal alteration of the shallow crust caused by the intrusion of dikes and sills of the Columbia River Basalt Province. It is estimated that heating and hydrothermal alteration of sediments rich in organic matter and carbonates around the dikes and sills releases 18 Gt of greenhouse gases (CH_4_ and CO_2_). Furthermore, hydrothermal δ^18^O depletion of rocks around dikes covers 500–600 km^3^, which, when scaled to the total CRB province constitutes 31,000 km^3^ of low-δ^18^O rocks. These volumes of crust depleted in δ^18^O are sufficient to explain the abundant low-δ^18^O magmas in eastern Oregon and western Idaho. This work also demonstrates that the width and magnitude of δ^18^O depletion around dikes can identify them as feeders. Given this, we here interpret Paleoproterozoic dikes in Karelia with the world’s lowest δ^18^O depletions (−27.8‰) as feeders to the coeval large igneous province aged 2.2–2.4 Ga that operated under the Snowball Earth glaciation conditions.

## Introduction

Intrusion and extrusion of basalts in large igneous provinces (LIP) such as the Siberian Traps, the Central Atlantic Magmatic Province (CAMP) and the Karoo have long been correlated with significant climate effects and extinction pulses due to the release of CO_2_, Hg, and SO_2_^1–5^. For example, the Permo-Triassic warming and mass extinction was proposed to be due to the release of greenhouse gases by Siberian Traps magmas themselves and/or by thermal release of CO_2_ and CH_4_ from early Paleozoic coal beds that were heated by the associated basalts and dikes^5^. To better understand the effects that LIPs have on the climate and environment, a better knowledge of how and to what degree the shallow continental crust is altered by meteoric waters in volcanically active areas is paramount.

Meteoric-hydrothermal cells generated by shallow cooling dikes have the potential to volatilize organics by either oxidation to CO_2_, or by reduction to CH_4_. Such effects were observed in geologically-obvious hydrothermal explosion pipes above shallowly emplaced basaltic lavas in the Karoo, CAMP, and Siberian LIPs, all of which are associated with climatic disruption^6–8^. However, the role of water is more cryptic in outcrop, and evidence of hydrothermal modification of crust commonly requires careful isotopic analysis to search for the tell-tale δ^18^O depletion that fingerprints the involvement of meteoric water, both in continents and also in submarine environments.

The role and extent of impact and influence of low-δ^18^O meteoric water on normal to high δ^18^O (supra)crustal rocks have been a subject of prior discussion in the 1970s to late 1980s^9^, but because many labs have since shifted to the analysis of unaltered phenocrysts, the effects of whole-rock modifications of volcanically active areas has become a topic of subordinate interest. A need thus exists for the study of the oxygen isotope impact of cooling magma bodies in a large igneous province setting.

In this study, we use oxygen and hydrogen isotope measurements and fluid circulation models to quantify the degree of hydrothermal metamorphism of the shallow continental crust produced by the dike swarms that fed the ~16 Ma Columbia River Flood Basalt Province (CRB), which will form a critical basis for understanding both the origin of low-δ^18^O rhyolites that erupted as part of the CRB (e.g.^10,11^) and the potential impact on climate from degassing of crustal rocks.

## Geologic background

**Figure 1:**
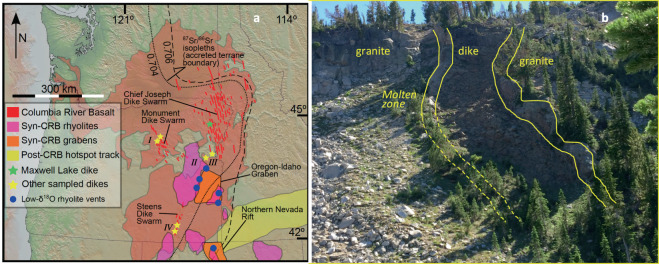
(**a**) Columbia River Basalt province showing dikes, lava flows and silicic centers coeval with the CRBs and younger (modified after^11^) as well as sample locations. See Table S1 for chemical and isotopic values. Roman numerals indicate dike groups. **(b)** Field photograph of the Maxwell Lake dike where basalt melted granitic wall rock of Wallowa Batholith (see Fig. S1 for detailed view of the contact).

The Columbia River Flood Basalt Province (CRB; Fig. 1) is the youngest and one of the most well-studied LIPs in the world, and consists of as much as 210,000 km^3^ of basalt and perhaps 10,000 km^3^ of coeval rhyolites^11–14^. Past and recent high-precision dating suggests that 95% of the main phase of the CRB, represented by the Grand Ronde basaltic to andesitic lavas, erupted between 16.7 and 15.9 Ma^4,10^, suggesting a high rate of both eruption and intrusion. However, the emplacement of the CRB had, if at all, only a minor impact on climate and environment^3,5^.

Single lava flows extending 300 km (such as the Roza flow, with average volume of individual lavas ~1300 km^3^ ^14^) document the high intensity of the eruptions and likely large magma supply rates. Still more dramatically and relevant for this study is the Wapshilla Ridge Member (WRM) of the Grande Ronde Formation, which is with an estimated volume of ~40,000 km^3^ ^3^ the largest package of lava flows in the CRB. It is bracketed by overlapping zircon ashfall dates of 16.288 ± 0.039 Ma and 16.254 ± 0.034 Ma by^4^, which highlights its high eruption rate.

These basalts are associated with extensive dike swarms (Fig. 1), which may have played a role in the small climatic impact of the CRB through heating and contact metamorphism of the crust^4,5,8^. While many of the dikes are buried under the thick CRB lavas, recent uplift of the province in the east has exposed inner workings of the crustal dike system (down to ~2 km depth^15–17^), allowing an insight into dike-crust interactions^18–20^. This unique setting shows that dikes intruded into a variety of rock types including earlier erupted CRB lavas, granitic batholiths, and Paleozoic metasedimentary rocks that often contain organic-rich shale and carbonates. Dikes range in thickness from 1 to >100 m, although the distribution strongly peaks at ~8 m, and larger structures likely record multiple reoccupations^15,16^. Based on the number of segments exhibiting partial melt in host rocks^16^, an estimated 3% of all known dikes served as feeders for lava flows and long-lived transport was probably highly localized along strike^19^. As they would have been potentially active for much longer, these feeder dikes likely caused larger degrees of thermal metamorphism and contact melting in country rocks than dead-end dikes that did not reach the surface^18–20^.

We analyzed 27 samples from 22 different dikes, as well as a detailed profile from wall rock to dike for the 10 m-wide Maxwell Lake dike (total of 23 samples) of the Columbia River Flood Basalt Province for their oxygen isotope compositions (Fig. 1). The Maxwell Lake dike has been geochemically correlated with the WRM and therefore the peak of CRB activity, and cross-cuts tonalitic granites of the Jurassic Wallowa Batholith in Oregon, USA^15,18,19^. Previous studies of the degree of dehydration partial melting of the wall rocks^18^ and apatite and zircon thermochronology of those same wall rocks^19^, have demonstrated that country rock was heated to as much as 900 °C at the dike contact and to 100 °C above background temperatures at distances of up to 100 m from the dike. Thermal modeling further suggested that the dike supported a continuous flow of magma for ~4–7 years, and therefore likely fed WRM flows^18–20^. Previous thermal models have, however, neglected the potential role of circulating hydrothermal fluids in cooling the dike, and the degree to which these fluids hydrothermally alter the country rock is unclear, both of these unknowns we resolve and emphasize with this study.

## Methods

### Isotope geochemistry

The 27 samples from small and large dikes of the CRB (Fig. 1) were analyzed for oxygen isotopes using laser fluorination and hydrogen isotopes + water using TCEA at the University of Oregon following published methodology (Appendix, Methods, all data are relative to VSMOW, errors are ±0.1‰ for δ^18^O, 1–3‰ for δD and ±0.05 wt% for H_2_O). For these samples, we relied on groundmass that record the effects of water-rock interaction and exchange oxygen easily. The same techniques for oxygen isotope measurements have been applied to the 23 samples from the profile across the Maxwell Lake dike and wall rock granite. Nine of these samples were previously studied for U-He ages^19^ while the other 14 samples were newly collected by us. Here, we measured the O isotopic composition of plagioclase and groundmass, two phases that nearly identically record the effects of water-rock interaction with comparable isotope fractionations^9^. In addition to plagioclase, we also analyzed other phases prone to aqueous alteration: magnetite, biotite and quenched melt (fine grained groundmass with glass), as well as quartz, pyroxene and amphibole - materials that are alteration-resistant and thus should reflect the original (unaltered magmatic) δ^18^O values.

XRF analyses for studied dikes were also obtained (see Appendix Table S1). Finally, we dated zircon grains (LA-ICPMS at the University of Bern, Switzerland) from samples CRB-60 (dike) and CRB-69 (granite) that define the contact melting zone of the Maxwell Lake dike, hoping that they would reflect the age of the dike. Unfortunately, no juvenile zircon of CRB age were found (Appendix Fig. S5, Table S3); instead, they are similar to published ages of other parts of the Wallowa batholith (125.6 ± 0.6 Ma^21^).

### Modeling

Modeling of hydrothermal fluid flow around a vertical cooling dike with a magma flow and hydrothermal circulation was done using the MUFITS program^22^, http://www.mufits.imec.msu.ru, which is a non-commercial reservoir simulator package, and is used for analysis of non-isothermal multiphase multicomponent flows in porous media (see Appendix for detailed methods and equations solved). In order to account for the heat from the flowing magma, we added point sources in computational domain cells located within a dike. Point sources were active for several years and then as magma flow stops, convective cooling of the dike occurs due to water circulation. Oxygen isotope exchange between fluid and country rock is calculated in a separate module that solves an advection-diffusion-reaction equation for known velocity and temperature fields. The exchange reaction is taken in Arrhenius form with a temperature-dependent reaction rate^23,24^. Further details on parameters used and oxygen isotope fractionation factors for rocks and water are given in the Appendix.

## Results

### Oxygen and hydrogen isotopes in the dikes and intruded crust

**Figure 2:**
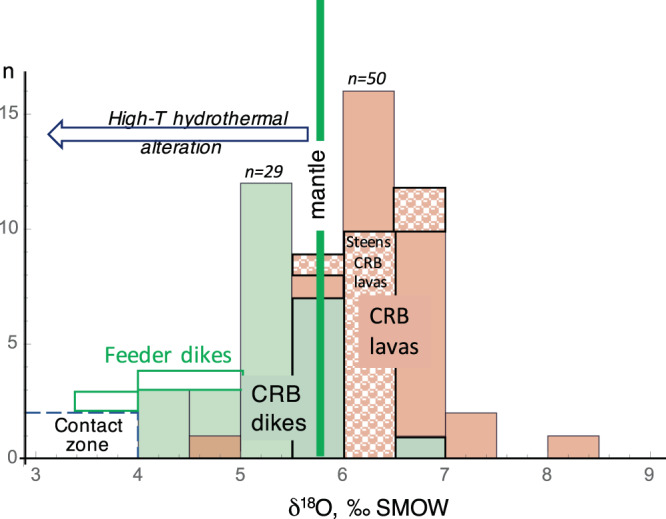
Comparison of δ^18^O values of dikes and lavas from the Columbia River Basalt province. Steen lavas represent early erupted primitive CRBs. The lower-δ^18^O values of dikes is explained by the effects of syn- to post-intrusive hydrothermal alteration by heated groundwater flow. The Maxwell Lake dike is outlined in green with country rocks (dashed blue) near the contact achieving the lowest δ^18^O values. Data for dikes is in the Appendix Tables. Oxygen isotope data for CRB lavas are from literature^11,25–27^ and were often obtained in the same lab (reported in^11,27^).

Oxygen and H isotope data, water content and XRF element concentrations are presented in appendix Tables S1-S2. Laser-ablation zircon U-Pb ages are shown in appendix Table S3. Whole-rock oxygen isotope compositions of CRB dikes are 1–2‰ lower on average than the δ^18^O of CRB lava flows (Fig. 2). The latter were quenched on the surface quickly without interaction with groundwater. The total magnitude of downward δ^18^O shift in dikes is even greater when considering that many of the CRB lavas are originally ~1‰ higher in δ^18^O than the mantle^25–27^.

The lowering in δ^18^O values in the dikes is considered a clear sign of high-temperature alteration by meteoric water, as weathering and secondary hydration increase (not decrease) the δ^18^O of altered material. This is because the isotopic fractionation factor between rock and water is greater than 16‰ (rock minus water) at temperatures lower than 90–125 °C (Appendix Fig. S4). Furthermore, meteoric water δ^18^O in the region in the mid-Miocene was likely heavier than the modern value of −11 to −14‰ on account of the then warmer climate^8^. Therefore, low temperature (≤100 °C) rock alteration (e.g. surface weathering effects) would have resulted in δ^18^O values much higher than is observed. This implies that our observed low-δ^18^O signal in the CRB-dikes (Fig. 2) is a product of high-temperature processes, associated with their intrusion and crystallization. We suspect that cooling of the dikes in a water-rich matrix drove a “self-inflicted” hydrothermal alteration during their prolonged syn-plutonic cooling that caused the low-δ^18^O values. The spatial extent of alteration is also in agreement with the previously-observed partial melting and resetting of He thermochronological clocks in apatite and zircon of the Maxwell Lake dike^18,19^, but extended to much smaller dike systems.

The hydrogen isotope (δD) of groundmass of the studied dikes also have meteoric, low-δD signatures of −128 ± 5‰ VSMOW, and dikes are hydrated to 1.2 wt% water on average (Table S1). As secondary alteration processes, such as weathering, can also lower δD values, we here document this low-δD data, but we do not rely on them for interpretation. They are likely a combination of a hydrothermal alteration signal from CRB times and later cold hydration.

**Figure 3:**
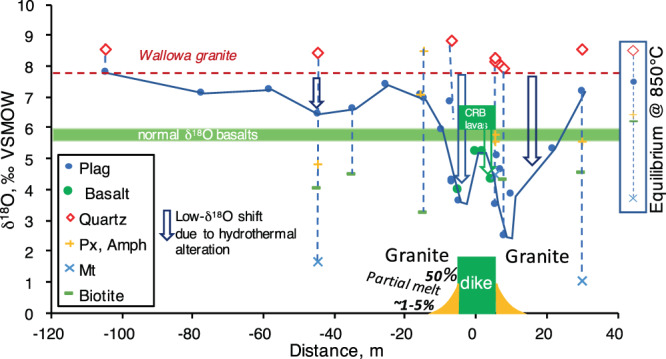
Oxygen isotope profile across the Maxwell Lake dike, the partially molten contact zone, and the tonalitic granites of Wallowa Batholith. Notice that the contact zone is the most depleted in δ^18^O signifying the strongest hydrothermal alteration in both basalt and melted country rocks. The zone of alteration extends several tens of meters in either direction. Quartz in granite is minimally affected, while easier to alter groundmass, plagioclase and other minerals are lowered and display large Δ^18^O_Qz-mineral_ fractionations due to effects of fluid flow and hydrothermal alteration. Oxygen isotopic compositions of minerals in equilibrium at high-T (850 °C) are given on the right, signifying the starting point prior to the meteoric-hydrothermal alteration event.

For the Maxwell Lake dike, we profiled this hydrothermal system in detail with a O isotope transect of both dike material and country rock across both sides of the dike (Fig. 3). The zone of low-δ^18^O alteration as documented by plagioclase and groundmass extends on both sides of the contact to distances of at least 50 meters. The lowest δ^18^O values of 2.5–4‰ are observed in both the partially molten and quenched granite and in the dike itself in the vicinity of the contacts on either side. More distant areas from the contacts have non-monotonically increasing δ^18^O in plagioclase, corresponding to variably disturbed Δ^18^O_quartz-plagioclase_ values. It is noteworthy that quartz, an alteration resistant mineral, shows no signs of lowering in δ^18^O, even in the partially molten zone near the contact (Fig. 3). Additional analyses of amphibole, pyroxene, biotite and magnetite returned δ^18^O values between magmatic values in equilibrium with quartz, and secondary values reflecting chloritization and oxidation by hydrothermal fluids. The δD value of the dike center is very low at −144‰ with 1.7 wt% water (Table S1). Oxygen isotope values of plagioclase and groundmass are used to monitor δ^18^O values of alteration intensity, which is shown in Fig. 3.

### Modeling

**Figure 4:**
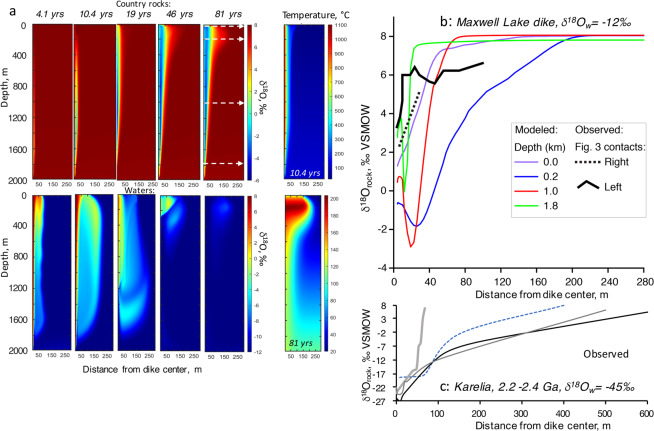
Numerical modeling of heat transfer and oxygen isotope exchange associated with a representative CRB feeder dike in water-saturated country rocks, see Figs. S2–5 in the Appendix for other results and movie files. Magma flow in the 10 m wide dike for 7 years is followed by cooling for 150 years, that matches data in Fig. 3 and the error envelope of U-He thermochronology modeling^19^, see also Fig. 5. Water is drawn from the surface, i.e. from the right side of the model. Hydrothermal flow in porous country rocks is induced and develops steep vertical flow features around the contact; transient vapor-water transitions at different times result in different stream functions and degrees of alteration at different depths and distances as heat advects and conducts. **(a)** Evolution of δ^18^O values of country rocks (upper panels), water (lower panels), and temperature at indicated times. Water is shifted up and rocks down in δ^18^O as a result of alteration. Figure is generated using Matlab version 2019a. https://www.mathworks.com/help/matlab/ref/movie.html. **(b)** Final δ^18^O depletions in rocks plotted vs. distance upon completion of fluid flow and cooling of the system (thin solid curves) and measured δ^18^O profile (dashed lines, from left and right sides of Maxwell Lake feeder dike, see Fig. 3). There is a significant vertical difference in δ^18^O level of the final depletion vs depth. This is due to time-integrated fluid flow through each area of the country rocks. Sampled depths are shown by dashed horizontal lines in **(a)**. **(c)** Observed δ^18^O depletions in rocks plotted vs distance for 2.2–2.4 Ga dikes in Karelia that interacted with -45‰ syn-glacial meteoric water (data from^32^). The Appendix and Fig. 5 present results of simulations in other initial and boundary conditions (profiles and movies), including duration of magma flow that ranges from 2 weeks to 7 years. Short magma flow durations result in a smaller magnitude of δ^18^O depletion. As the majority of CRB dikes are not feeders and have shorter magma flow, they exhibit smaller δ^18^O depletion (see Fig. 2).

In Fig. 4 we present results of modeling porous fluid flow which produces hydrothermal alteration and δ^18^O depletion around a cooling dike, performed using the MUFITS software^22^. We assumed a magma temperature of 1140 °C, a dike thickness of 10 m, and we varied the duration of flow (1–10 years) (cf. 18–20]) as well as the porosity and permeability of water-saturated country rocks (assuming spatially and temporally uniform host rock properties). Searching these parameters, we found a good match between previous observations of the dike environment and a permeability of K = 10^−13^ m^2^ and a porosity of 3%, corresponding to microfracture permeability in the host granite.

Modeling was able to reproduce the overall width of the δ^18^O depletion zone which extends ~150 meters from the contact, including the oscillating δ^18^O values with distance from the dike documented in Fig. 3, which are explained by a transient vapor phase behavior near the contact. Immediately after dike intrusion, magma heats and drives ambient meteoric water, some of which is converted to steam, away from the contact. During active magma flow within the dike, this initial behavior transitions into a convection cell of heated groundwater in the vicinity of the dike (Fig. 4, movies in Appendix), with upwelling of hot water near the dike-rock interface and down flow which draws in fresh low-δ^18^O meteoric water in the far field. This convective flow with upward flow along the contact then persists for 105 years after dike intrusion. Where rock temperatures are high, isotope exchange with meteoric water takes place leading to significant depletion of host rocks in heavy oxygen isotopes, particularly in the feldspar crystals (Figs. 3, 4). The fluid flow is also affected by liquid water to steam phase transitions which happen at an intermediate distance from the dike contact, affecting time integrated fluid flow and details of δ^18^O vs distance relationships. Steam in shallow conditions carries less molar oxygen than heated water, affecting molar oxygen flux. Finally, upon cooling of basalt in the dike below brittle-ductile transition, heated meteoric waters will penetrate inward and flow upward, causing alteration of the basalt to low-δ^18^O values (Fig. 3), though this process is not included in our models.

**Figure 5:**
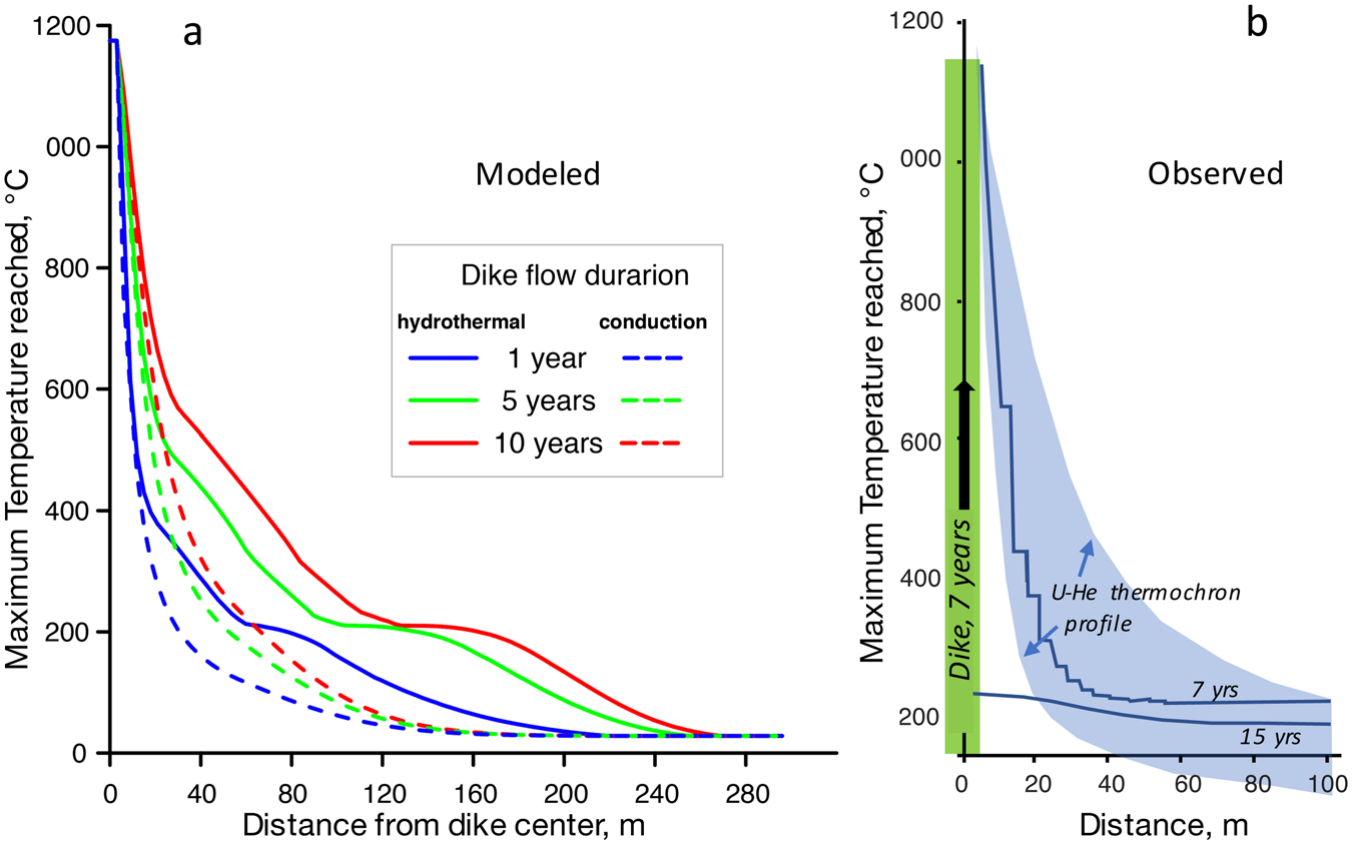
(**a**) Maximum temperature reached in simulations in convective runs (solid lines) vs identical conductive runs (dashed lines). Country rock permeability is 10^−13^ m^2^, porosity is 3% corresponding to an open fracture network. (**b**) comparison of simulations with T-distance profile (68% confidence intervals) from inversion of U-Th-He thermochronometric ages for zircon and apatite around the Maxwell Lake dike^19^, best matching 7 yr duration of magma flow in the dike.

The modeled convective cooling regime of the dike was matched with the temperature-distance profile constrained by Karlstrom *et al*.^19^, who performed a Bayesian Markov-Chain Monte Carlo inversion of U-Th-He zircon and apatite reset ages consisting of s>10^6^ individual simulations for various parameter combinations to derive the 68% confidence intervals shown in Fig. 5. Given the large width of the thermal influence zone extending ~100 m, these researchers had to assume thermal conductivities larger than those for pure conduction. Our advection-diffusion modeling to match O-isotopic observations suggests that heated water convection rather than conduction dominated near-dike heat transport. Figures 4–5 compare hydrothermal modeling results with the measured O isotopic compositions.

We further observe that the models predict the overall width and magnitude of δ^18^O depletion around the dike on timescales of magma flow and subsequent cooling at a permeability in the 10^−13^ m^2^ range, which generates fluid flow rates of many meters per year. This is within the permeability observed for MORB (10^−11^ to 10^−14^ m^2^ ^28^,). The model result also predicts something that is not measured in the field: a relationship between lowering of δ^18^O and distance from the dike that is variable with depth (Fig. 4). As the permeability in the model did not vary, this observation results from variable fluid fluxes undergoing water-vapor phase transition, and their time-integrated trajectories. Modeling provides a testable hypothesis to investigate δ^18^O variations over a 1 km vertical length of dike as well as the spatial extent of maximum depletion away from the dike, including its degree and isotopic sign changes. We consider the results of such modeling as both reassuring in that it mechanistically explains our observations, and in that it is possible to achieve the observed depletion of oxygen in and around the dikes on timescales of their cooling.

Published temperature-time histories in the vicinity of Maxwell Lake dike^18–20^ were based on an assumption of conductive heat transport in country rocks without hydrothermal effects, although the reset of He-apatite ages at greater distances than expected for conduction with normal thermal conductivity values (2–3 W/mC) was noticed^19^. Different parameterizations in the above models of dike heating and treatment of melting and release of latent heat largely agreed that heating of country rocks by introducing basalt occurred for around 1–7 years, raising the contact temperature to ~850–950 °C, enough to cause ~50% melting of granite at the contact. Upon cessation of the flow in the dike, the models predict that 0.6–0.7 years are required to cool both, the basalt and granitic partial melts near the contact to granite solidus (725 °C; Fig. S2). Conductive and convective cooling of the dikes are compared on Fig. 5a, demonstrating wider thermal influence on the country rocks when meteoric water circulation is involved. Thermal and hydrothermal effects associated with dikes with different duration of magma flow are further compared in the Supplementary Movies in the Appendix.

Hydrothermal alteration processes and modeling inferred for the Maxwell Lake Dike are likely applicable to the other studied, smaller dikes presented here (Figs. 1, 2**)**. Their low-δ^18^O values demonstrate that ambient fluids entered the dikes after the dikes cooled to the brittle-ductile transition at around 400–500 °C and that hydrologic pressure injected low-δ^18^O water into hot rocks. Based on the modeling, fluid flow direction around the dike can be visualized in three stages: (1) flow away from the heating contact during intrusion, (2) upward parallel to the contact upon hydrological recharge, and (3) outward and into the dike upon it cooling below the brittle-ductile transition.

## Discussion and Implications

### Hydrothermal systems around cooling dikes in LIPs

We provide the first documentation of syn-plutonic hydrothermal alteration around a dike in the CRB. The pattern of decreasing alteration with distance from the intrusion is analogous to the two-dimensional “bull’s eye” patterns of hydrothermal alteration described for many larger intrusive systems around the world^29,30^. In all such systems, the center of the intrusion serves as a heat source which drives hydrothermal convection in the solidified outer intrusion and in the surrounding country rock. The central, hotter areas facilitate greater lowering of rock δ^18^O, because high temperatures produce lower Δ^18^O_mineral-water_ fractionation factors (Appendix Fig. S4) and because they stay hot and active for longer (Fig. 4**)**, allowing more complete equilibration. The hot center and melted contact zones are shifted closest to the altering low-δ^18^O meteoric water values. The existence of such hydrothermal systems around dikes in the CRB has also been inferred by Buchan *et al*.^31^ from changes in remanent magnetization of country rocks.

The Maxwell Lake dike is unusual compared to most other basaltic dikes because it is associated with a very large altered area (~100 m in diameter) relative to a fairly small (<10 m) intrusion, and because it was altered to lower δ^18^O values than most other dikes (Figs. 2–3**)**. This is because, as a long-lived feeder, it drove a hotter and longer-lived hydrothermal system than other dikes that represent shorter-lived and less voluminous intrusive events. These long durations of activity imply very large magma volumes (associated Wapshilla Ridge flows are up to 5000 km^3^ or more and are probably almost uniquely associated with LIP activity^3,15^). The kind of pervasive low-δ^18^O alteration in and around a relatively small dike seen at Maxwell Lake and other dikes is therefore diagnostic of LIP activity in the geologic record, where dikes are commonly much thicker.

For example, the world’s greatest δ^18^O depletions (down to −27.8‰) are found in and around thick (10–80 m) dikes of 2.2–2.4 Ga age in Karelia, Russia and in the Scourie area of Scotland^32,33^. These studies suggest that these dikes are likely also feeders to voluminous surface flows, given the large spatial extent of hydrothermal alterations they caused. It also connects them directly to the 2.4–2.2 Ga LIP in the Baltic Shield^33^ that are also contemporaneous to the severe Snowball earth glaciations in the Paleoproterozoic with low δ^18^O surface waters down to −40‰. The oxygen isotope alteration pattern around basaltic dikes (found here) can thus serve to detect the presence of LIP-scale eruptive activity that is otherwise hidden, or eroded away especially in the Precambrian. It further identifies that these dikes had shallow emplacement even if they cut crystalline basement. We (and other researchers) previously were unable to explain the width of δ^18^O depletions in Karelia given that the width of even the thickest of the dikes in the Khitostrov area (~80 m) are incapable to such an extensive δ^18^O change unless they are feeders.

Our work may find further support in explaining extreme hydrothermal events in other, and especially submarine LIPs. For example, Beier *et al*.^34^ recently documented that ~10 Ma submarine Azores Plateau in the Central Northern Atlantic Province exhibit features of extreme hydrothermal alteration during submarine igneous plateau formation, which drastically changed the compositions of the igneous crust.

### Regional low-δ^18^O crust modification and connection to low-δ^18^O rhyolite production

Our observation that syn-CRB dikes have induced “self-inflicted” hydrothermal alteration is important to understand the degree of lowering of δ^18^O in the upper crust in volcanically active areas, and by extension in submarine LIP and MORB environments as well^34^. Rifting and closely-spaced heat sources such as dikes and sills likely lead to significant hydrothermal alteration similar to what we document in this work. Generally, this study shows that LIP generate abundant meteoric hydrothermal episodes and wide-spread hydrothermal modification of the upper crust, or shallow submarine crust. Contact-hydrothermal δ^18^O depletion of rocks around dikes is 500–600 km^3^, which scaled to the CRB footprint constitutes 31,000 km^3^ of low-δ^18^O rocks. Collectively, these volumes of crustal δ^18^O depletion are sufficient to explain the abundant low-δ^18^O magmas in eastern Oregon and western Idaho^10,11,35,36^, where the most extension and diking takes place. Upon re-melting and assimilation during subsequent phases of magmatism, these hydrothermally altered rocks may become low-δ^18^O magmas especially if magmas generally follow the same already established magma plumbing systems, in which rocks are additionally preheated to ease assimilation efficiency. Such a scenario seems to have occurred to the south of the Maxwell Lake dike at the Oregon-Idaho graben (Fig. 1), where syn-volcanic normal faults appear to have concentrated basalt diking and therefore the production of low-δ^18^O magmas through this multi-step process^11^. The total volume of low-δ^18^O magmas formed during collision of the CRB plume with the North American plate has not yet been established as exposures are lacking. However, it is likely that the ~10,000 km^3^ of low-δ^18^O rhyolites in the post-CRB Snake River Plain hotspot track^35,36^ were produced by this process, especially if a feeder dike system was repeatedly reactivated.

### Thermogenic gas production and limited climate impact of the CRBs

If we assume that the small non-eruptive dikes shown in Fig. 2 all have induced thermal metamorphism with various associated aspects of hydrothermal alteration just several diameters of the dike around the dikes themselves (e.g. Fig. 5), then the extent of hydrothermal alteration in the CRB province is estimated to affect 500–600 km^3^ shallow continental crust. This estimate is based on the cumulative length of dikes, assuming that each feeder dike affected country rocks 150 meters in each direction from the contacts, and that dead-end dikes just affected country rocks by 30 meters at average dike thickness of 8 m with vertical extent of 3 km^16^.

We choose the dike number density exposed in the Chief Joseph Dike Swarm in the Wallowa Batholith and assumed that dikes are intruded in metasedimentary (15%), volcanic (53%) and plutonic (32%) igneous crust in proportions based on Morriss *et al*.^16^. These authors independently estimated that up to 10–100 km^3^ of metasediments were affected by the heat of the dikes. The total volume of CRB Group basalt is estimated at 210,000 km^3^ ^37^. Using these, we obtain ~600 km^3^ of rocks affected by hydrothermal alteration, and assuming that 2 wt% of organic matter is present on average in metasedimentary country rocks, along with a high-end estimate of 0.5 wt% in the dominant plutonic and volcanic country rocks, volatilization of this as CO_2_ will release only 18 Gt of greenhouse CO_2_ gas. This is a small number compared to the 1000–3000 Gt of CO_2_ released by the 210,000 km^3^ of CRB magmas themselves (assuming 0.2–0.5 wt% CO_2_^38^). Even if the dike-affected area is scaled to the 210,000 km^2^ footprint of CRB, the thermal and hydrothermal release of greenhouse gases due to oxidation of soil and country rock organics will constitute only about a third of magmatic CO_2_ release. The lack of known extinction pulses associated with CRBs^8^, which intruded primarily through and on top of the volcanic and plutonic basements, as opposed to the Siberian Traps and Karoo^6,7^, which erupted into organics-rich sedimentary rocks, may be related to this small initial amount of organics available for volatilization by the CRB, despite our observation of effects of hydrothermal metamorphism.

## Supplementary Information

Supplementary Information.

Supplementary Information 2.

Supplementary Information 3.

Supplementary Information 4.

Supplementary Information 5.

Supplementary Information 6.

Supplementary Information 7.
