# Supplementary material for: Pervasive Hydrothermal Events Associated with Large Igneous Provinces Documented by the Columbia River Basaltic Province
Source: Sci Rep. 2020 Jun 23;10:10206. doi: 10.1038/s41598-020-67226-9 (PMC7311473; doi:10.1038/s41598-020-67226-9)
Supplement: Supplementary file 1 — Supplementary Information. [file 41598_2020_67226_MOESM1_ESM.docx]

**Title Page for the Supplementary Information:**

**Pervasive Hydrothermal Events Associated with Large Igneous Provinces Documented by the Columbia River Basaltic Province**

Bindeman I.N*. ^1,2^, Greber N.D. ^2,4^, Melnik O.E.^3,5^, Artyomova A.S.^3,5^,

Utkin I.S.^5^, Karlstrom L.^1^, and Colon D.P.^1,2^

^1^Earth Sciences, University of Oregon, Eugene, OR, USA;

^2^ Department of Earth Sciences, University of Geneva, Switzerland

^3^Fersman Mineralogical Museum, Moscow, Russia;

^4^Institute of Geological Sciences, University of Bern, Switzerland;

^5^Institute of Mechanics, Moscow State University, Russia

*****corresponding author, Bindeman@uoregon.edu

The Supplementary material consists of:

***Supplementary Tables:***

Table S1 Chemical composition, oxygen and hydrogen isotopes, and bulk water content of CRB dikes sampled and studied

Table S2. Oxygen isotope composition of rocks and minerals across the Maxwell Lake dike contact

Table S3. U-Pb Ages of zircons in Wallowa Batholith (sample CRB69) and partially molten zone (sample CRB60) determined by LA-ICP-MS

***Supplementary Figures:***

Fig. S1 Sampled Contact between Maxwell Lake dike basalt and Wallowa tonalitic granite, field photos

Fig. S2 Screenshot of movies that simulate hydrothermal flow, oxygen isotope exchange

and temperature around Maxwell Lake dike with magma flow lasting for 1, 5, and 10 years.

Magma flow in movies start at 50 years. Blue-steped line represents maximum temperature

reached during the entire duration of the simulation. See Movie File for development of the process. Movies are generated using Matlab software version R2019a.

Figure S3. Exploration of δ^18^O depletion around 1m and 10 m thick dikes with different duration of magma flow shown

Figure S4 Mineral and rock –water oxygen isotopic fractionation factors used in numerical simulations and explanation of isotopic effects

Figure S5 LA-ICP-MS Concordia Diagram for zircons in samples CRB-60 and CRB-69 with partial melt around Maxwell Lake dike

***Supplementary Movies:***

1_yr.mov: Magma flow for 1 year in the dike, followed by cooling and hydrothermal exchange

5_yr.mov: Magma flow for 5 years in the dike, followed by cooling and hydrothermal exchange

10_yr.mov: Magma flow for 10 years in the dike, followed by cooling and hydrothermal exchange

Movies showing magma flow in the dike for 1, 5, and 10 years, followed by continuous hydrothermal activity, cooling, and isotopic exchange. This is an example of simulations that were performed in these study for Fig. 4 in the main text of the paper. Movies are generated using Matlab software version R2019a. https://www.mathworks.com/help/matlab/ref/movie.html

***Supplementary Methods***

Analytical and numerical, text file
