# Supplementary material for: Pervasive Hydrothermal Events Associated with Large Igneous Provinces Documented by the Columbia River Basaltic Province
Source: Sci Rep. 2020 Jun 23;10:10206. doi: 10.1038/s41598-020-67226-9 (PMC7311473; doi:10.1038/s41598-020-67226-9)
Supplement: Supplementary file 5 — Supplementary Information 5. [file 41598_2020_67226_MOESM5_ESM.docx]

Appendix : METHODS

*Analytical methods*

The δ^18^O and total H_2_O and δD measurements were conducted on a gas source MAT 253 isotope ratio mass spectrometer (IRMS) at the University of Oregon using TCEA and laser fluorination methods described in Bindeman et al. (2012) and Martin et al. (2017) and Loewen et al (2019).

Analyses of total H_2_O and δD of water in glass were conducted with a high temperature conversion elemental analyzer (TC/EA) interfaced with the MAT 253 IRMS. For TCEA-δD measurements we used between 2 and 6 mg of glass and fine groundmass depending on its water content to make the H_2_ and DH peaks of the same magnitude as compared to the reference gas peaks. Loaded 50-150 um size fractions were dried at 130°overnight in a vacuum oven to remove any adsorbed water. Samples were introduced into the TC/EA in sealed Ag-foil capsules using an autosampler, where standards were interspersed with the unknown. Samples fall into a glassy carbon reactor inside a furnace held at 1450°C under continuous He flow. Upon melting, volatiles in the sample are liberated, which undergo a pyrolysis reaction that generates H_2_ and CO gas. These gases are separated by gas chromatography and are transported to an open split by the He carrier gas. A reference gas of known composition is also introduced into the open split to monitor instrument stability. Both the sample gas and the reference gas are introduced to the IRMS from the open split. Mica standards USGS57 (biotite), USGS58 (muscovite) (Qi et al., 2017) are used for calibration and are analyzed 4-5 times throughout each analytical session for H_2_O. The δD data are additionally calibrated with USGS water standards (W-62001, VSMOW, USGS-47, and USGS-53). δD is determined with a precision 1-3‰, and water is determined with a precision of ±0.02-0.05wt.%.

For single chip δ^18^O analyses, 1-2 mg of single chunks of glass were loaded into a sample fluorination chamber. We used a CO_2_ laser fluorination line connected to the MAT 253 IRMS. The laser chamber was pretreated with BrF_5_ reagent until acceptable blanks were achieved, except when we run reactive samples. In this case we used an airlock sample chamber where samples are introduced one by one in prefluorinated chamber. Samples then were lased in the presence of BrF_5_. To liberate O_2_, the sample gas was purified by a series of liquid nitrogen traps and a Hg diffusion pump to get rid of excess of disproportionated F_2_. The O_2_ was converted to CO_2_ by a small carbon-platinum rod converter. Yields were recorded and for most samples were better than 90%, for aggressively pretreated samples with yields of 70%, there is no correlation of δ^18^O vs yield. CO_2_ gas was introduced to the mass spectrometer for analysis. Samples were analyzed together with the standards in the same sample plug and data were normalized to their nominal VSMOW values, using San Carlos Olivine (δ^18^O =5.2±0.1‰ and an internal Gore Mountain garnet standard, UOG (δ^18^O =6.52‰). Error on δ^18^O are ±0.1‰. All O and H Isotope compositions are expressed in delta notation relative to Vienna Standard Mean Ocean Water (VSMOW).

*Numerical Methods*

In order to simulate the flow and isotope patterns generated by magma intrusion we use MUFITS software to obtain temperature and velocity fields and a specially developed module for isotope transport and exchange reaction. MUFITS (http://www.mufits.imec.msu.ru) is a non-commercial reservoir simulator package capable for analysis of non-isothermal multiphase multicomponent flows in porous media. It solves mass (1) and energy (2) conservation laws together with Darcy flow equations (3). We assume pure water as a porous fluid.

$\frac{\partial}{\partial t}\left( m\sum_{i=1}^{2} \rho_{i}s_{i} \right)+div\left( \sum_{i=1}^{2} \rho_{i}s_{i}w_{i} \right)=0$ (1)

$\frac{\partial}{\partial t}\left( m\sum_{i=1}^{2} \rho_{i}e_{i}s_{i}+(1-m)\rho_{s}e_{s} \right)+div\left( \sum_{i=1}^{2} \rho_{i}s_{i}h_{i}w_{i}-\lambda grad T \right)=0$ (2)

$w_{i}=-K\frac{f_{i}}{\mu_{i}}\left( grad p+\rho_{i}\boldsymbol{g} \right)$ (3)

Here *i*={water, vapor}, *ρ_i_* are the densities of pure substances, *s_i_* are water and vapour saturations (*s_1_*+*s­_2_*=1), *m* is the porosity, *w_i_* are Darcy velocities, *e_i_* are internal energies, index “s” corresponds to the skeleton of the porous media, *h_i_* are the enthalpies, λ is the averaged thermal conductivity, T is the temperature, K is the permeability, *f_i_* and *µ_i_* are the relative permeabilities and viscosities, *p* is the pressure and ***g*** is the gravity acceleration vector.

The temperature, density and water saturation are specified as a function of pressure and enthalpy. This allows to have stable numerical method and ovoid difficulties around the critical point of water.

The dyke is assumed impermeable and, thus, only energy equation is solved in this domain. Simulation domain is plain 2D rectangle with the left boundary located at the dyke central line, height *H* = 2000 m and width *W* = 300 m. At the upper boundary z=H constant pressure and temperature are assumed if the upward velocity is negative (fluid enters the domain) or no boundary condition is specified for outflowing fluid. In this case the values from the nearest cell are transferred to the boundary. Remaining boundaries are kept adiabatic and no-flux condition is applied. Temperature gradient of 40 ^o^C/km and hydrostatic pressure distribution are specified as initial conditions.

Isotope transport and exchange are simulated by the solution of the following set of equations:

$mX_{f}\frac{\partial C_{f}}{\partial t}=-F-mX_{f}\nu\cdot grad(C_{f})+div\left[ mX_{f}D_{f} grad(C_{f}) \right]$ (4)

$\left( 1-m \right)X_{s}\frac{\partial C_{s}}{\partial t}=+F$ (5)

Here *C_f_* and *C_s_* are the concentrations of ^18^O, $X_{f}$ and $X_{s}$ are the number of moles of oxygen $({}^{18}{O+{}^{16}{O)}}$ per cubic meter in the fluid and rock, respectively, $D_{f}$ is the isotope diffusion coefficient, $F$ is the exchange reaction rate of ${}^{18}O$ between the fluid and the rock defined as $F=\gamma(\alpha C_{f}-C_{s})$. Here *γ* is the reaction rate constant$\gamma=S_{0}A_{0}exp(-\frac{E_{a}}{RT})$ , *S_0_* is the surface area per unit volume, *A_0_* is the pre-exponent factor, *E_a_* is the activation energy, *R* is the universal gas constant. Isotopic fractionation factor α is defined as:

${10}^{3}\ln\left( \alpha\right)=\frac{{10}^{6}A}{T^{2}}+B$ (6)

Here A and B are constants defined in Fig. A4 in Appendix

We assume that initially the rocks are saturated with meteoric water with δ^18^O =- 12 ‰. For the rocks δ^18^O =+8 ‰. There reaction rate is extremely small due to low temperatures. At no-flux boundaries concentration gradients are assumed to be equal to zero, at the upper boundary if the water enters the domain δ^18^O is specified. Otherwise the isotopic concentration is taken from the nearest cell to the boundary.

Parameters used in the simulations.

| Parameter | Notation | Value |
| --- | --- | --- |
| Porosity | $m$ | $3\%$ |
| Permeability | $K$ | 10^-13^ m^2^ |
| Rock permeability | $\lambda_{s}$ | $2 W\cdot m^{-1}\cdot K^{-1}$ |
| Rock heat capacity | $c_{s}$ | $1.1 kJ\cdot{kg}^{-1}\cdot K^{-1}$ |
| Rock density | $\rho_{s}$ | $2600 kg\cdot m^{-3}$ |
| Magma temperature | $T_{m}$ | $1150 C$ |
| Diffusion coefficients | $D$_f_ | $5\times{10}^{-8} m^{2}\cdot s^{-1}$ |
| Moles of oxygen in m^3^ of water | $X_{f}$ | $62500 mol\cdot m^{-3}$ |
| Moles of oxygen in m^3^ of rocks | $X_{s}$ | $82500 mol\cdot m^{-3}$ |
| Constant in isotopic fractionation factor | $A$ | $2.78$ |
| Constant in isotopic fractionation factor | $B$ | $-2.89$ |
| Specific surface area | $S_{0}$ | ${{10}^{3} m}^{-1}$ |
| Pre-exponential factor | $A_{0}$ | $4.72\times{10}^{-4} {mol\cdot m}^{-2}\cdot s^{-1}$ |
| Activation energy | $E_{a}$ | ${43.472 kJ\cdot mol}^{-1}$ |
| Universal gas constant | $R$ | ${8.3144 J\cdot mol}^{-1}\cdot K^{-1}$ |
