# Supplementary material for: Pervasive Hydrothermal Events Associated with Large Igneous Provinces Documented by the Columbia River Basaltic Province
Source: Sci Rep. 2020 Jun 23;10:10206. doi: 10.1038/s41598-020-67226-9 (PMC7311473; doi:10.1038/s41598-020-67226-9)
Supplement: Supplementary file 6 — Supplementary Information 6. [file 41598_2020_67226_MOESM6_ESM.pptx]

## Slide 1
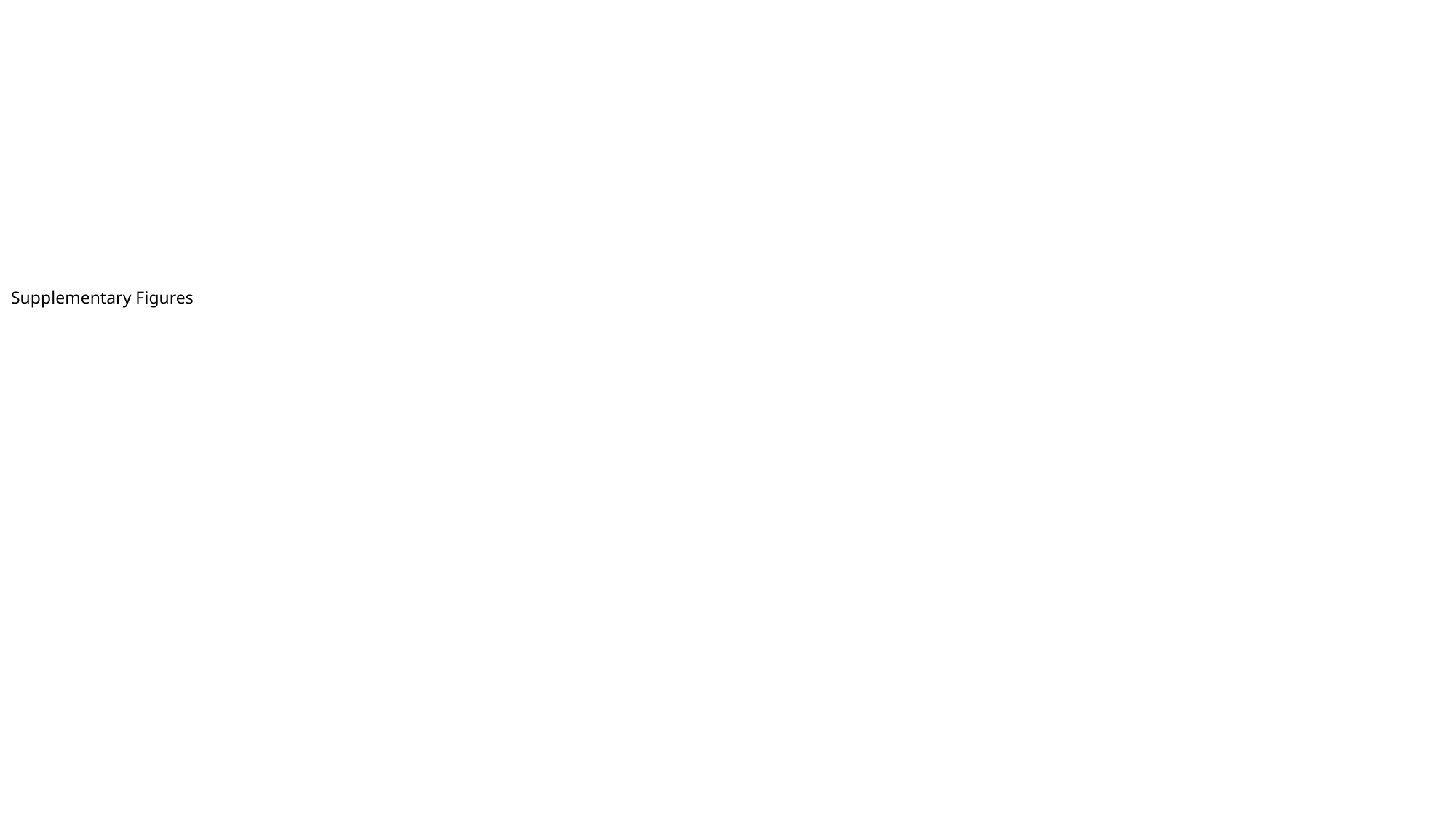

# Supplementary Figures

## Slide 2
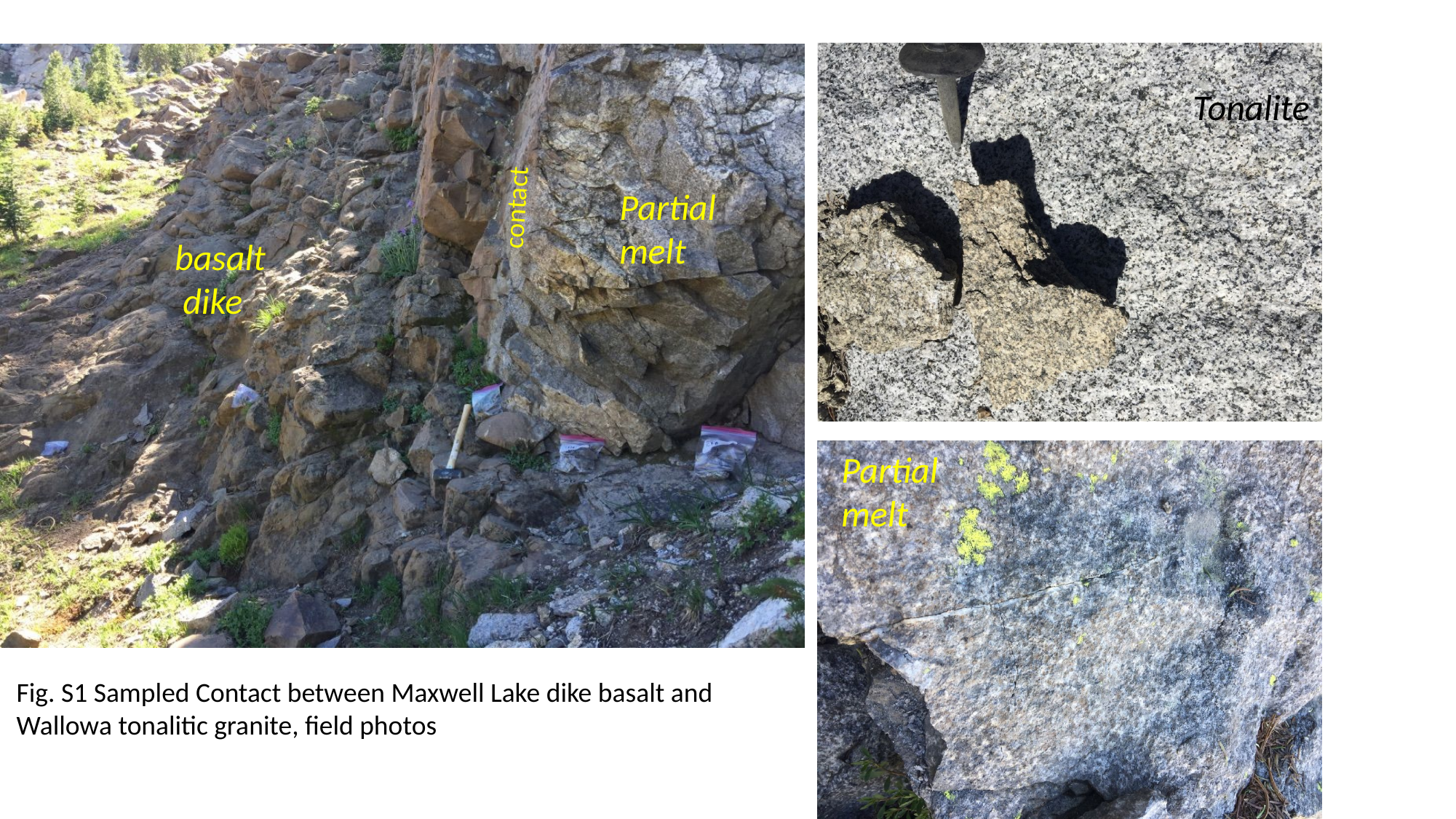

Tonalite
Partial
melt
contact
basalt
 dike
Partial
melt
Fig. S1 Sampled Contact between Maxwell Lake dike basalt and
Wallowa tonalitic granite, field photos

## Slide 3
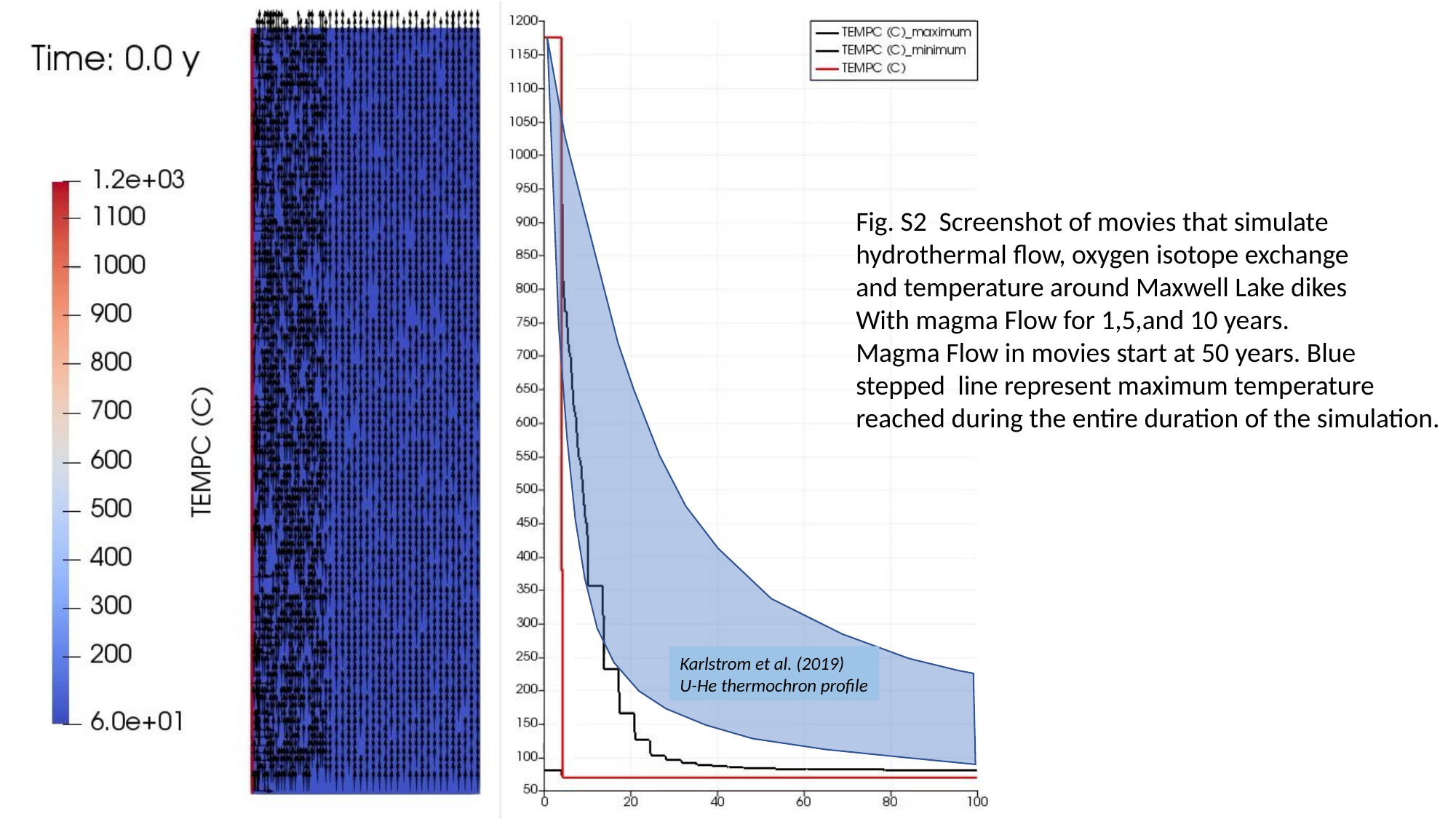

Fig. S2 Screenshot of movies that simulate
hydrothermal flow, oxygen isotope exchange
and temperature around Maxwell Lake dikes
With magma Flow for 1,5,and 10 years.
Magma Flow in movies start at 50 years. Blue
stepped line represent maximum temperature
reached during the entire duration of the simulation.
Karlstrom et al. (2019)
U-He thermochron profile

## Slide 4
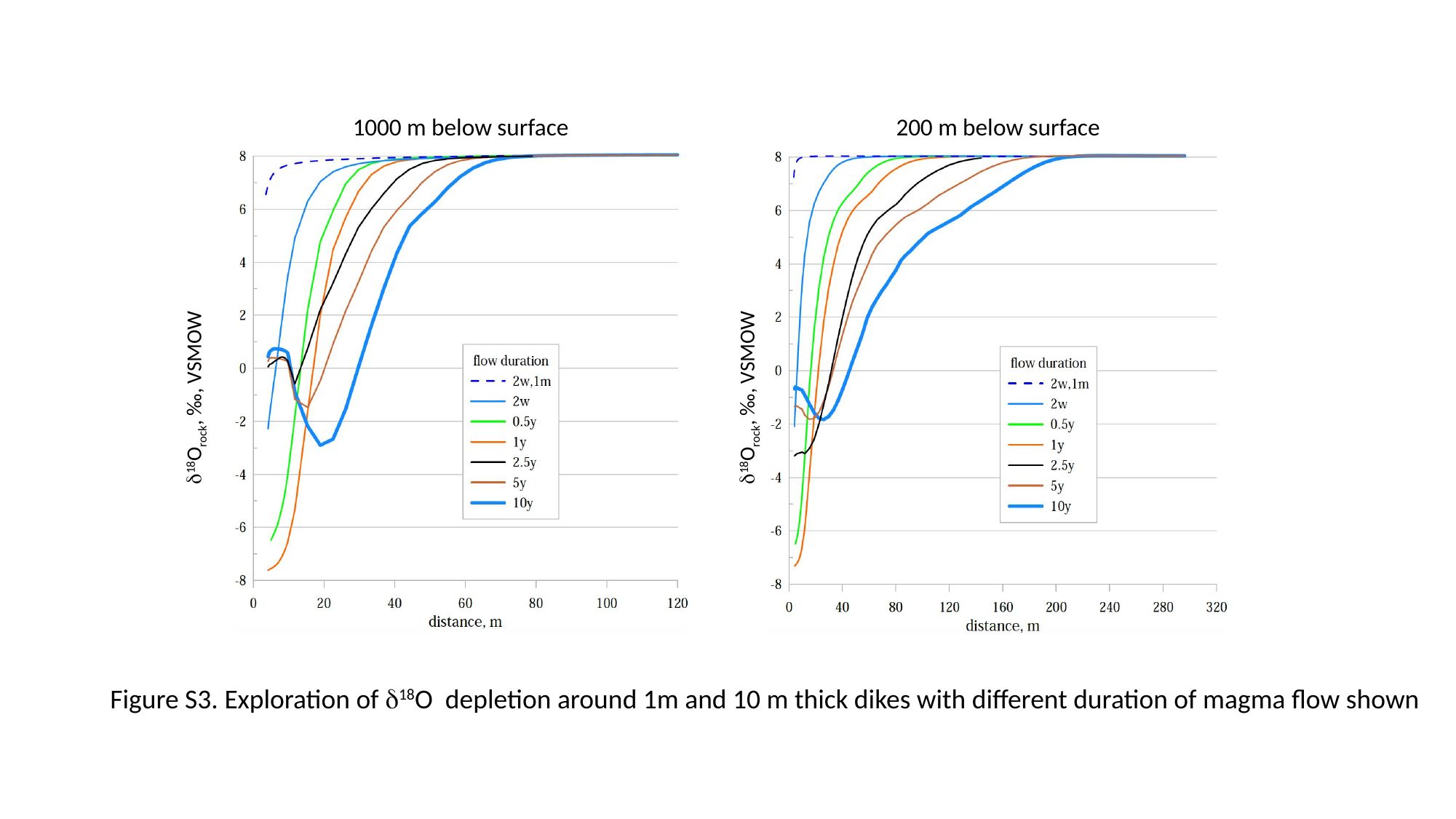

1000 m below surface
200 m below surface
d18Orock, ‰, VSMOW
d18Orock, ‰, VSMOW
Figure S3. Exploration of d18O depletion around 1m and 10 m thick dikes with different duration of magma flow shown

## Slide 5
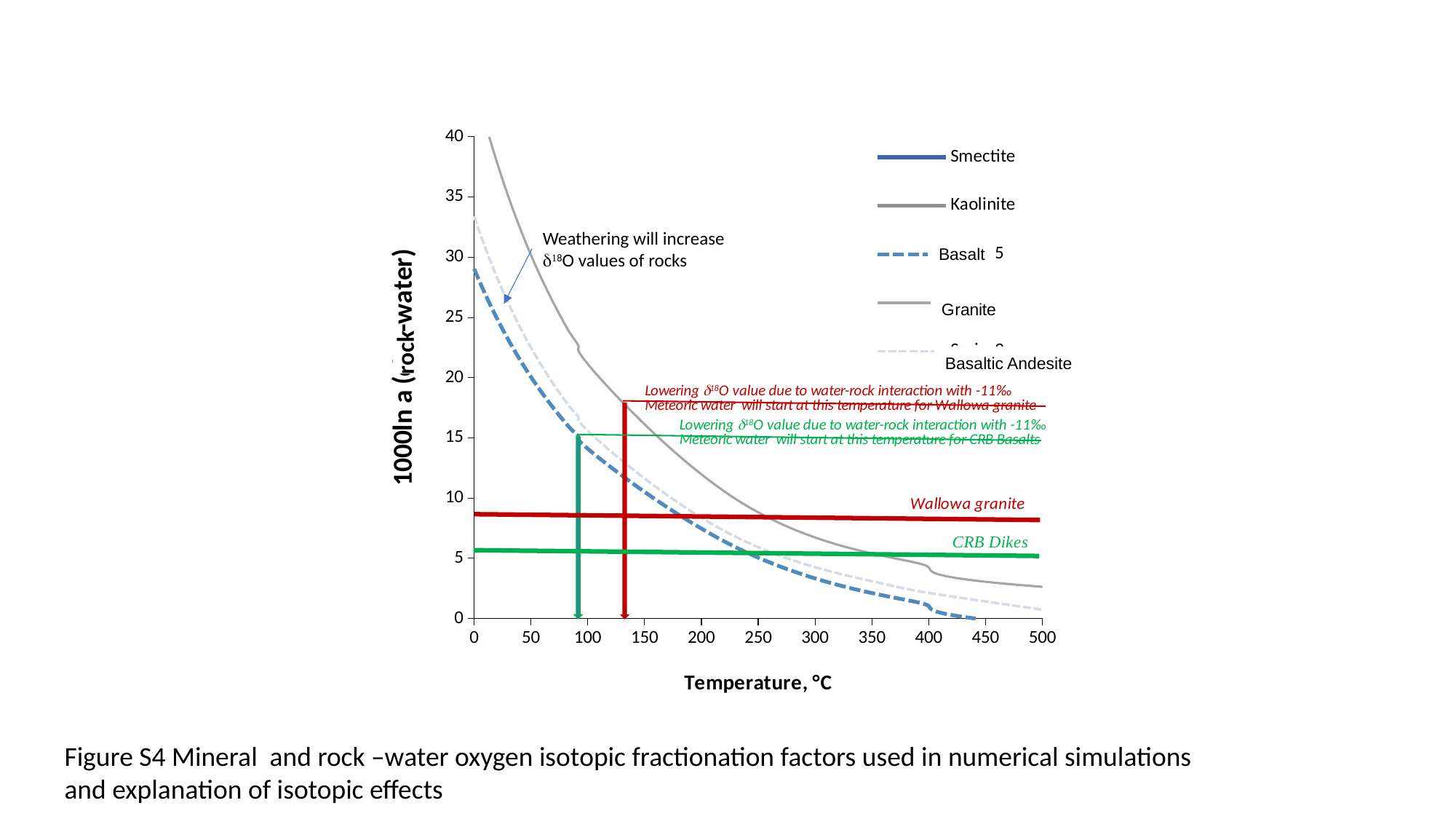

### Chart
| Category | | | | | |
|---|---|---|---|---|---|Weathering will increase
d18O values of rocks
Basalt
rock
Basaltic Andesite
Figure S4 Mineral and rock –water oxygen isotopic fractionation factors used in numerical simulations
and explanation of isotopic effects

## Slide 6
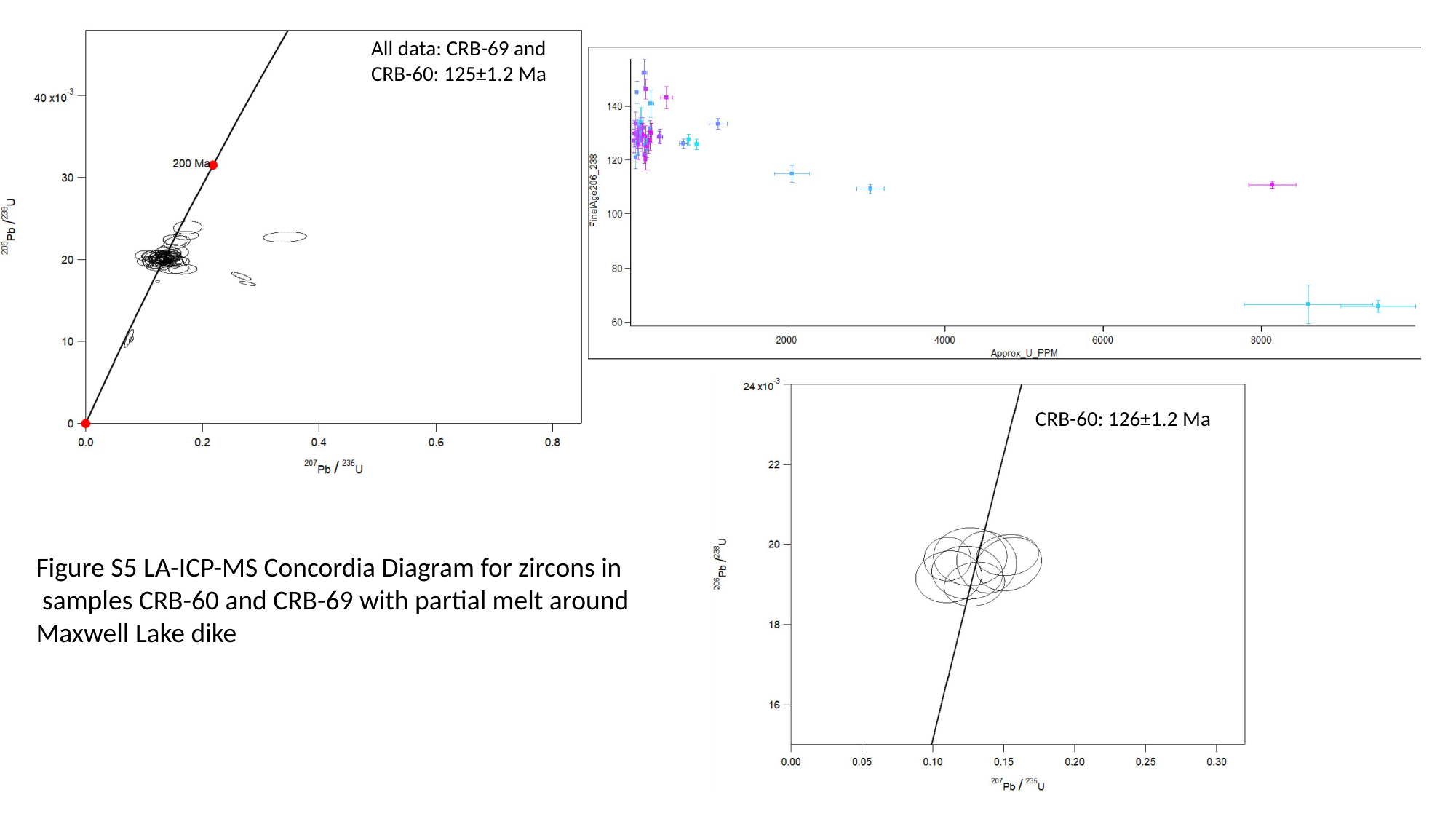

All data: CRB-69 and
CRB-60: 125±1.2 Ma
CRB-60: 126±1.2 Ma
Figure S5 LA-ICP-MS Concordia Diagram for zircons in
 samples CRB-60 and CRB-69 with partial melt around
Maxwell Lake dike
